# Intraoral Approach Through the Nasal Floor for Surgical Removal of Inverted Mesiodens: Protocol and Case Series

**DOI:** 10.3390/jcm13247831

**Published:** 2024-12-22

**Authors:** Horatiu Urechescu, Ancuta Banu, Felicia Streian, Florin Urtila, Cristiana Cuzic, Stefania Dinu, Marius Pricop

**Affiliations:** 1 Department of Oral and Maxillo-Facial Surgery, Faculty of Dental Medicine, University of Medicine and Pharmacy “Victor Babes”, 300041 Timisoara, Romania; urechescu.horatiu@umft.ro (H.U.); banu.ancuta@umft.ro (A.B.); urtila.florin@umft.ro (F.U.); pricop.marius@umft.ro (M.P.); 2 Department of Prosthodontics, Faculty of Dental Medicine, University of Medicine and Pharmacy “Victor Babes”, 300041 Timisoara, Romania; pricop.cristiana@umft.ro; 3 Department of Pediatric Dentistry, Faculty of Dental Medicine, University of Medicine and Pharmacy “Victor Babes”, 300041 Timisoara, Romania; dinu.stefania@umft.ro; 4 Pediatric Dentistry Research Center, Faculty of Dental Medicine, University of Medicine and Pharmacy “Victor Babes”, 300041 Timisoara, Romania

**Keywords:** inverted mesiodens, nasal floor, CBCT

## Abstract

**Background/Objectives:** The most common form of supernumerary teeth is represented by the mesiodens. Very often, they are impacted, usually palatially, but can be found buccally or between the roots of the permanent central incisors. Their position can be normal, inclined, or inverted. In the possible case where the impacted mesiodens crown is oriented upwards towards the nasal cavity, they are called inverted mesiodens. The inverted mesiodens are mainly asymptomatic, and the main diagnostic methods are radiological, especially CBCT. **Methods:** This paper presents the intraoral approach through the nasal floor as a very reliable method for the surgical removal of inverted mesiodens. We report a protocol, including the diagnosis process, criteria for choosing this surgical approach, and description of the surgical procedure. Preoperative CBCT is mandatory for diagnosis and choice of the surgical approach. This is chosen based on measurements on the cross-sectional view of the CBCT investigation and is in compliance with our criteria related to the appropriate surgical approach. **Results:** Using this approach, the mesiodens were extracted without intraoperative or postoperative complications or accidents, and operating times were relatively short. **Conclusions:** The intraoral approach through the nasal floor for surgical removal of inverted mesiodens satisfies all the conditions to be a predictable, safe, and time-efficient technique. It has its limitations, mainly regarding the surgical skills of the operating team. The cone–beam computed tomography (CBCT) has a fundamental role in the diagnosis and treatment of included mesiodens.

## 1. Introduction

The presence of supernumerary teeth in addition to the normal formula is a dental anomaly that affects the maxilla more than the mandible, mostly the premaxilla region, and is referred to as mesiodens. Data from the recent literature show a prevalence of mesiodens of 5.04% that is more common in males [1]. Under certain conditions, the mesiodens can erupt and be visible on the dental arch and may have a fully formed root. However, if the crown of the mesiodens is oriented superiorly; it is unlikely that it will erupt intraorally. Inverted conical mesiodens can be accidentally found just below the nasal cavity. In this situation, they are referred to as being in an inverted position [2,3].

The etiology of supernumerary teeth is not yet very clear. Studies found in the literature attribute the development of these dental entities to several factors: the phylogenetic reversion or atavism, the hyperactivity of the dental lamina, or a combination of genetic factors. The clinical findings in the case of supernumerary teeth are often absent. If present, they are represented by dental crowding, delayed eruption or impaction of normal dentition, spacing disturbances, development of follicular cysts, neuralgic manifestations, and occasionally, eruption into the nasal cavity. Studies in the literature show that 75% of the mesiodens are impacted [4,5].

Due to the absence of clinical signs, in most cases, an impacted mesiodens is detected based on 2D radiographs such as orthopantomographs (OPGs), occlusal or periapical, and, in some cases, even lateral cephalometric radiographs. Currently, cone–beam computed tomography (CBCT) is a valuable tool that can significantly improve the accuracy of diagnoses by providing 3D cross-sectional images [6]. Studies from the literature carried out in the early mixed dentition stage show that although present, the mesiodens does not erupt in 79–91% of the cases. In 7–20% of the cases analyzed, its presence is observed on routine radiographs without being associated with another pathology [7,8]. This fact confirms that the age of the mixed dentition is the period in which impacted mesiodens are most commonly diagnosed and also highlights the importance of routine dental radiographs.

The surgical approach used to remove an impacted mesiodens is chosen based on the position of the mesiodens in the alveolar bone. Palatal positioning of the mesiodens relative to the upper central incisors is the most commonly encountered situation. In this case, a palatal approach is preferred [9]. Unfortunately, the main disadvantages of this approach are represented by a low level of accessibility associated with an increased risk of damage to the nasopalatine nerve. In order to avoid these deficiencies, one can opt for using the buccal approach, despite the increased risk of damage to the roots of the central incisors [10].

It can be considered that the chances of complications resulting from the surgical removal of the impacted mesiodens are proportional to its positioning. Close proximity to the apices of the neighboring teeth, especially in the superior and deep positionings in the alveolar bone, significantly increases the risk of complications. These can also be prevented using a method that provides a good surgical view, and this is the main advantage in the case of the traditional buccal approach. The palatal approach can avoid periodontal problems in the maxillary anterior teeth that could result from the buccal approach. Postoperative swelling is also reduced compared to the buccal approach. Excessive osteotomy and long operating times should be avoided because they may reduce blood perfusion and pulpal vitality of the adjacent teeth [11]. In 2011, a transoral approach to intranasal teeth, a surgical method called “the modified maxillary vestibular approach with subperiosteal intranasal dissection”, was proposed. According to the authors, using this method prevents excessive osteotomy and limits the possible complications of buccal or palatal approach methods. Another advantage of this method is the operating time, which is significantly shorter compared to traditional methods [12]. In addition, an endoscopically assisted transnasal method can be used for the removal of mesiodens erupting into the nasal floor [13].

The location and morphology of inverted mesiodens will affect the choice of treatment, but to date, there is no generally accepted surgical approach for the extraction of inverted mesiodens.

## 2. Materials and Methods

This report presents the intraoral approach through the nasal floor as a very reliable option for extracting inverted mesiodens. This method was first described by Sammartino et al. in 2011 [12], but the main constraints were mainly related to the damage to the nasal mucosa and misplacing the mesiodens into the nasal cavity with the risk of aspiration and airway obstruction.

We report our protocol, including the diagnosis process, criteria for choosing this surgical approach, and description of the surgical procedure. This study was approved for publication by the ethics committee of Timisoara Municipal Emergency Hospital where this study was conducted (E-6388/04.12.24).

### 2.1. Diagnosis Process

**Figure 1:**
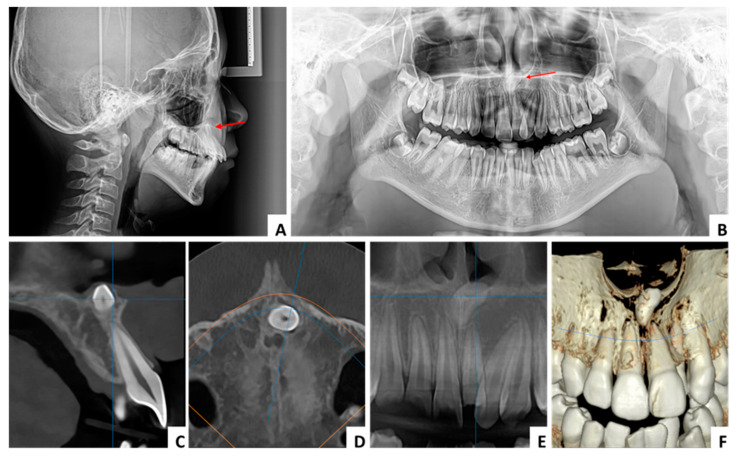
Radiological diagnostic methods—conventional 2D radiographs vs. CBCT in a case of included mesiodens: (**A**) lateral cephalometric—mesiodens highlighted by the red arrow, (**B**) OPGs—esiodens highlighted by the red arrow, (**C**) CBCT cross-sectional view, (**D**) CBCT axial view, (**E**) CBCT panorama view, (**F**) CBCT 3D reconstruction.

The inverted mesiodens are mainly asymptomatic; in some cases, a diastema can be associated. The main diagnostic methods are radiological, especially CBCT. They can be detected also by conventional imaging techniques used in dental practice, such as OPGs, but in this case, there are some serious limitations regarding the lack of clarity in the midline region (Figure 1). Preoperative CBCT is mandatory in our protocol regarding included mesiodens. It provides an accurate 3D localization, position, and shape of the impacted teeth and allows us to choose the appropriate surgical procedure (Table 1).

### 2.2. Choice of Surgical Approach

The main criteria taken into account for the determination of the surgical approach are represented by the shortest linear distance to the mesiodens, a good surgical field, the prevention of extensive osteotomy, the protection of neighboring teeth, the prevention of neurovascular injury to the nasopalatine nerve, and avoiding prolonged operation time and limited postoperative discomfort for the patient. In the case of inverted mesiodens breaching the nasal floor (submucosally or covered with bone), the intraoral approach through the nasal floor satisfies all these conditions (Table 2).

### 2.3. Nasal Floor Approach Procedure

The patients require short-term hospitalization, and the surgical procedure is performed under general anesthesia (naso or oro tracheal intubation). Local anesthesia (articaine with epinephrine) is also performed for pain control and limited bleeding, thus providing a better visualization of the surgical field. A vestibular maxillary incision from the canine to the canine is made, followed by subperiosteal dissection and the elevation of a full-thickness flap up to the level of the ANS and piriform aperture. The nasal mucosa is elevated from the underlying bone of the nasal floor using periosteal elevators. At this point, care must be taken not to perforate the mucosa. This creates access to the anterior portion of the nasal floor. When necessary, bone from the nasal floor can be removed with rotary instruments under saline irrigation. The mesiodens are exposed, gently luxated, and removed through the nasal floor using extraction elevators. Bleeding control is achieved, and the surgical site is irrigated with saline solution. The mucoperiosteal flap is repositioned and sutured with a single layer of 4/0 rapid absorbable sutures. The follow up includes routine clinical and radiological examinations for the assessment of possible postoperative complications.

### 2.4. Case Series

**Figure 2:**
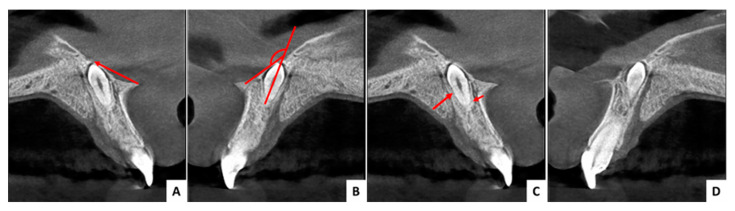
CBCT analysis: (**A**) distance from the ANS highlighted by the red arrow, (**B**) angle formed by the mesiodens axis with the nasal floor highlighted by the red lines, (**C**) distance to the mesiodens from the buccal and palatal aspects highlighted by red arrows, (**D**) position regarding the nasopalatine canal.

We report a series of cases to support the predictability of this surgical technique. In all cases, no clinical symptoms were recorded, and the inverted mesiodens were identified following routine radiographic examinations for dental or orthodontic purposes. All patients underwent a CBCT for diagnosis and choice of the surgical approach. The following parameters were determined on the cross-sectional view of the CBCT investigation: the angle formed by the mesiodens axis with the nasal floor, the distance from the ANS, the distance to the mesiodens from the buccal and palatal aspects, the relation with the nasal cavity (submucosally or covered with bone), and the position regarding the nasopalatine canal (Figure 2).

Taking into account all these parameters and the criteria for choosing the surgical technique, it was decided to use the nasal floor approach procedure for the surgical removal of the inverted mesiodens. All surgeries were performed by the same surgical team, consisting of a maxillofacial surgeon and two resident surgeons using the same surgical protocol. The postoperative medication consisted of antibiotics (Amoxicillin/clavulanic acid) and nonsteroidal anti-inflammatory drugs (Ibuprofen). The usual postoperative indications were given related to diet, oral hygiene, swelling, and, in one case, the patient was told to avoid smoking.

The patients were called for follow ups, and the last radiograph (OPG) that was recorded was dated three years after surgery.

## 3. Results

The mesiodens were extracted without damaging the roots of the adjacent teeth or the nasopalatine canal. No injury to the nasal mucosa was recorded. The operation time was approximately 30 min in all cases. No other intraoperative or postoperative complications or accidents occurred. None of the patients complained about bleeding, breathing, swelling, difficulties in feeding or maintaining oral hygiene, hypoesthesia, or postoperative pain.

One of the patients was female, aged 29, and the other two were male, aged 11 and 16. In one case, the angle formed by the mesiodens axis with the nasal floor was less than 90° (85.93°), and in the other two cases, it was greater (143.5° and 144.4°). The distance from the ANS was less than 10 mm in two cases (8.98 mm and 8.11 mm) and greater in one (13.22 mm). In all cases, the shortest distance to the mesiodens was from the buccal aspect, and the mesiodens were positioned in front of the nasopalatine canal. In two cases, the mesiodens were covered with bone, and in one case, they were positioned submucosally (Table 3).

### 3.1. Case 1 (Figure 3, Figure 4 and Figure 5)

**Figure 3:**
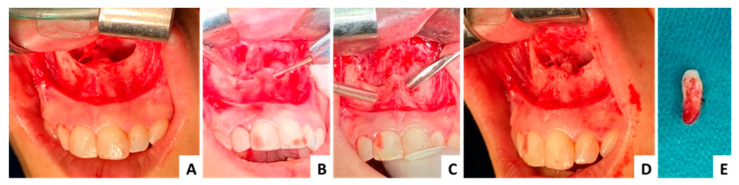
Case 1—clinical aspects: (**A**) elevation of the mucoperiosteal flap and nasal mucosa, (**B**) osteotomy with rotary instruments, (**C**) luxation and extraction of the mesiodens using extraction elevators, (**D**) intraoperative aspect after extraction, (**E**) extracted mesiodens.

**Figure 4:**
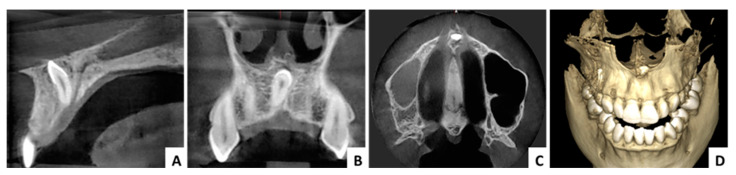
Case 1—radiological aspects: (**A**) CBCT cross-sectional view, (**B**) CBCT panorama view, (**C**) CBCT axial view, (**D**) CBCT 3D reconstruction.

**Figure 5:**
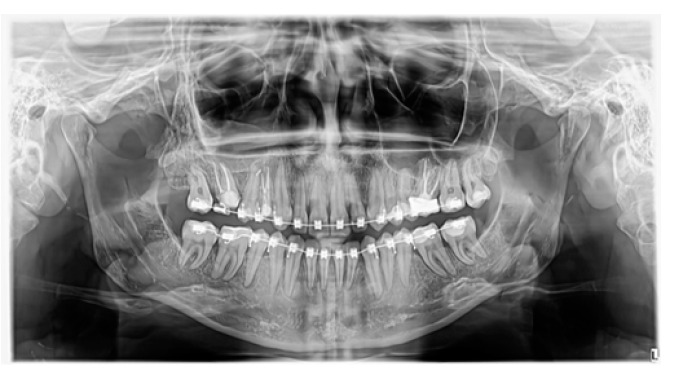
Case 1—follow-up OPG, three years after surgery.

The first case presented is of a female patient, 29 years old at the time of surgery, diagnosed with an impacted inverted mesiodens. The patient did not present any symptoms, the diagnosis was made following radiological investigations necessary for orthodontic treatment. The mesiodens was covered with bone which was removed with rotary instruments. No accidents or surgical complications were recorded (Figure 3, Figure 4 and Figure 5).

### 3.2. Case 2 (Figure 6, Figure 7 and Figure 8)

**Figure 6:**
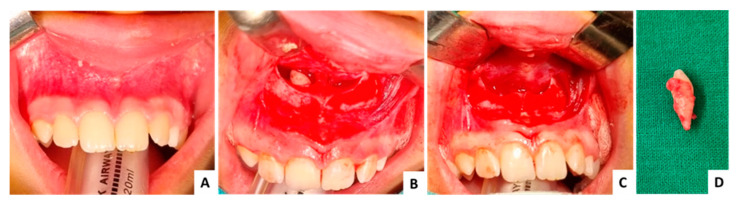
Case 2—clinical aspects: (**A**) initial clinical aspect, no symptoms recorded, (**B**) exposed mesiodens, (**C**) intraoperative aspect after extraction, (**D**) extracted mesiodens.

**Figure 7:**
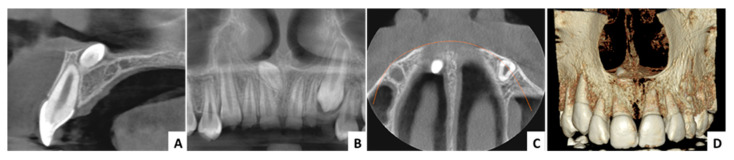
Case 2—radiological aspects: (**A**) CBCT cross-sectional view, (**B**)—CBCT panorama view, (**C**) CBCT axial view, (**D**) CBCT 3D reconstruction.

**Figure 8:**
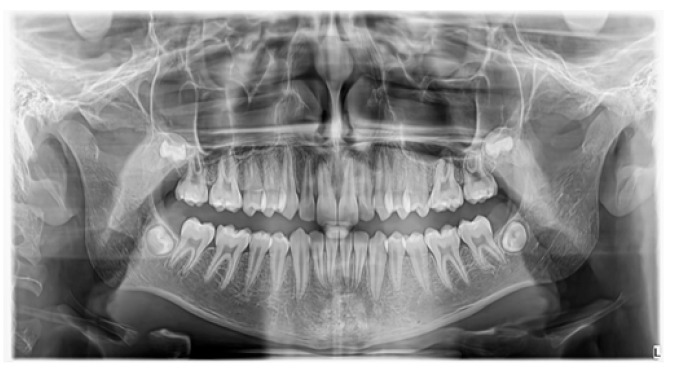
Case 2—follow-up OPG, three years after surgery.

The second case is of a male patient, 11 years old at the time of surgery, diagnosed with inverted included mesiodens following routine radiological investigations. The surgery was uneventful, with no complications (Figure 6, Figure 7 and Figure 8).

### 3.3. Case 3 (Figure 9, Figure 10 and Figure 11)

**Figure 9:**
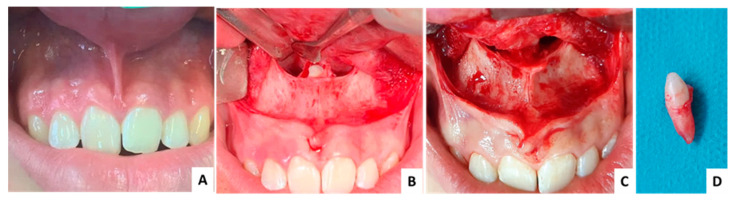
Case 3—clinical aspects: (**A**) initial clinical aspect, no symptoms recorded, (**B**) exposed mesiodens, (**C**) intraoperative aspect after extraction, (**D**) extracted mesiodens.

**Figure 10:**
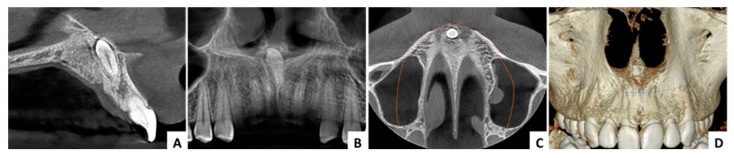
Case 3—radiological aspects: (**A**) CBCT cross-sectional view, (**B**) CBCT panorama view, (**C**) CBCT axial view, (**D**) CBCT 3D reconstruction.

**Figure 11:**
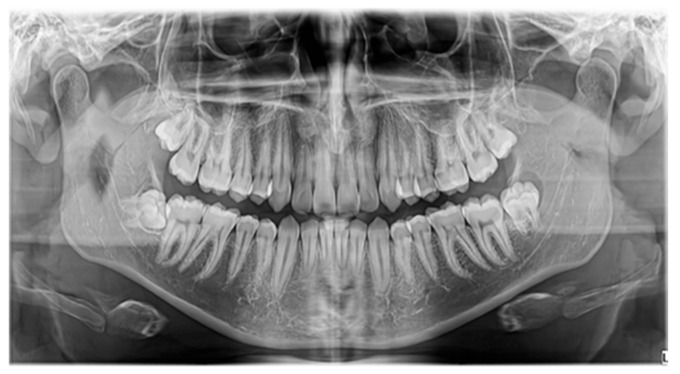
Case 3—follow-up OPG, three years after surgery.

The third case is also of a male patient, 16 years old at the time of surgery. The patient did not present any symptoms, the diagnosis was made following radiological investigations necessary for orthodontic treatment. The surgery was without accidents or complications (Figure 9, Figure 10 and Figure 11). 

In all cases, the control radiograph (OPG) taken approximately three years after the surgery did not show any long-term complications or abnormalities in maxillary development (Figure 5, Figure 8 and Figure 11).

## 4. Discussion

Data from the literature certify that among supernumerary teeth, the mesiodens are the most common [6,14,15]. In the majority of cases, they are impacted, usually palatially, but they can be found buccally or between the roots of the permanent central incisors. Their position can be normal, inclined, or inverted [6].

The most common complications related to the presence of mesiodens are those related to dental crowding, delayed eruption or impaction of normal dentition, spacing disturbances, root resorption of the adjacent teeth, and cystic lesion formation or other bone-destructive lesions [8,16,17]. Considering these complications, prophylactically surgical extraction is recommended [18,19,20].

Regarding the appropriate age for the surgical removal of the mesiodens, early studies found in the literature indicate a delay in surgical treatment until the roots of the permanent teeth are fully formed, indicating an age of 8–10 years. This recommendation was made to prevent intraoperative damage to the tooth germs of neighboring permanent teeth [21]. Recent studies indicate removal of the mesiodens at an early age at the time of detection, ideally by the age of 5–6 years. This can prevent the occurrence of complications related to the presence of the mesiodens [8].

If the mesiodens are oriented upwards, towards the nose, then we refer to them as inverted mesiodens. According to different studies, inverted mesiodens are reported to occur in 9 to 67% of cases [8,22,23]. They can develop towards the base of the nose or the nasal septum. In some situations, the inverted mesiodens can even be found in the nasal cavities. It is reported that they are found in contact with the nasal floor bone in 20.5% of cases [5].

For the diagnosis and treatment of impacted mesiodens, clinical and radiographical assessments are essential. Conventional 2D radiographs, such as orthopantomographs (OPGs), have some serious limitations regarding the lack of clarity in the midline region. Cone–beam computed tomography (CBCT) is an imaging technique that allows for an accurate 3D localization of impacted teeth and has an essential role in choosing the optimal surgical approach [24].

The endoscopically assisted transnasal approach is a useful method to remove inverted mesiodens found in the nasal cavities or for mesiodens located under the nasal mucosa [7,25].

The most common method used for extraction in the case of an included mesiodens mentioned in the literature is the palatal approach, followed by the buccal approach [26,27]. The traditional buccal approach provides an excellent surgical view. In the case of inverted mesiodens, using this method requires excessive osteotomy, and it may lead to an increase in intraoperative accidents and postoperative complications, such as damaging the roots of the permanent incisors. Another drawback of this approach is that excessive osteotomy may exacerbate postoperative swelling and pain. To prevent all this, the palatal approach is an alternative, but it has the disadvantage of a significant risk of damaging the nasopalatine nerve, especially when the mesiodens are positioned in front of the nasopalatine canal [10].

In 2011, Sammartino et al. presented a new method called the “modified maxillary vestibular approach with subperiosteal intranasal dissection”, which is very useful for surgical removal of inverted impacted mesiodens [12]. Using this approach, extensive osteotomy can be avoided in comparison with the palatal or the buccal approach. When necessary, osteotomy is limited to the area around the crown of the impacted tooth, and then the inverted mesiodens can be luxated in the direction of its implantation axis towards the nasal cavity [11]. Reducing intraoperative bleeding is also an important advantage of this technique, and this is due to the fact that the bone blood perfusion in the nasal floor is not as important as in the buccal plate, and thus we have less damage to the blood supply during surgery [28]. The nasal floor approach also provides a good direct view of the surgical site, and considering the minimal osteotomy needed and reduce bleeding, it can provide a short operating time. The approach proves to be effective and minimizes surgical trauma, leading to a significant decrease in postoperative swelling and pain. Studies found in the literature show that this approach offers the highest degree of patient satisfaction compared to the buccal or palatal approach. The low level of pain and swelling allows patients to return to their normal routine very soon after surgery [11].

The main constraints of the approach are mainly related to the damage to the nasal mucosa and misplacing the mesiodens into the nasal cavity with the risk of aspiration and airway obstruction [11]. For this reason, it seems that this method is contraindicated for dentists without advanced surgical experience. The approach is similar to that used in procedures, such as Le Fort I osteotomy or SARPE, which is why this method is indicated for maxillofacial surgeons with experience in orthognathic surgery [13].

For the predictability of treatment and limitations of intra- and postoperative complications, a precise protocol related to the diagnostic process and the choice of surgical approach must be applied in the case of an impacted mesiodens.

The criteria that must be taken into account are the shortest linear distance to the mesiodens, good surgical field, the prevention of extensive osteotomy, the protection of neighboring teeth, the prevention of neurovascular injury to the nasopalatine nerve, the avoidance of a prolonged operation time, and limited postoperative discomfort for the patient. To satisfy all these criteria, a thorough CBCT analysis must be performed. This analysis includes the following determinations: the angle formed by the mesiodens axis with the nasal floor, the distance from the ANS, the distance to mesiodens from the buccal and palatal aspects, the relation with the nasal cavity, and the position regarding the nasopalatine canal.

Previous studies in the literature have suggested the nasal floor approach, in cases where the angle formed by the mesiodens axis with the nasal floor, is less than 90° [27,29]. Other studies recommend a distance of up to 10 mm between the ANS and the mesiodens [10].

However, in our experience, it was not difficult to surgically remove inverted mesiodens through the nasal approach, even in cases where the angle formed by the mesiodens axis with the nasal floor was greater than 140° and the distance from the ANS was greater than 10 mm. Even in these situations, no intra- and postoperative complications or longer operating times were reported.

## 5. Conclusions

Within the limitations of this paper, the intraoral approach through the nasal floor for the surgical removal of inverted mesiodens satisfies all the conditions to be a predictable, safe, and time-efficient technique. Like any other surgical technique, it has its limitations, mainly regarding the surgical skills of the operating team and previous experience in orthognathic surgery being necessary. Used correctly, it can be a technique that greatly limits the risk of intra- and postoperative complications and leads to a significant decrease in postoperative swelling and pain. Nowadays, CBCT has a fundamental role in the diagnosis and treatment of included mesiodens. A precise protocol with clear criteria must be used to determine the type of approach in the case of included mesiodens.

## Figures and Tables

**Table 1:** The basic steps in the diagnosis process for impacted mesiodens.

Diagnosis Process	Observations
Clinical examination	The inverted mesiodens are mainly asymptomatic, but in some cases, they can cause midline displacement, diastema, rotation, dental crowding, delayed eruption or impaction of normal dentition, and spacing disturbances.
Conventional radiography (OPG, occlusal, periapical, lateral cephalometric radiographs)	In the case of conventional 2D radiographs, mainly OPGs, the structures outside the focal area may be covered by other structures and cannot be visualized. Also, precise localization in relation to other anatomical elements or neighboring teeth will require the use of an additional occlusal radiograph.
CBCT	A 3D radiographic imaging method is an excellent method of evaluation and diagnosis. It has a fundamental role in the precise localization of the mesiodens and a connection with the neighboring anatomical structures and teeth. Thus, it has an important role in choosing the appropriate surgical procedure.

**Table 2:** Determination of the surgical approach.

Criteria	Observations
Distance to the mesiodens	It is preferable to use the approach that offers the shortest distance to the included mesiodens.
Surgical field	The traditional buccal approach provides an excellent surgical view compared with the palatal approach. It has its limitations due to the fact that the included mesiodens are found predominantly on the palatal side. The nasal floor approach can also offer an optimal surgical view.
Osteotomy	Extensive osteotomy should be avoided because it may lead to an increase in intraoperative accidents and postoperative complications. It can also lead to severe swelling and pain in the days following surgery. Using the nasal floor approach, an extensive osteotomy is avoided.
Neighboring teeth	Included mesiodens mostly develop on the palatal side. Using the buccal approach involves a major risk of damaging the roots of neighboring teeth. For this reason, a palatal approach for surgical extraction is preferred.
Neurovascular injury	The palatal approach involves the risk of neurovascular damage to the nasopalatine nerve, especially when the mesiodens are positioned in front of the nasopalatine canal.
Operation time	Prolonged operation time may affect blood perfusion and vitality of neighboring teeth. Also, in these cases, postoperative swelling and pain will be significantly exacerbated.
Patient postoperative discomfort	None of the approaches used for the surgical removal of an inverted mesiodens guarantees a reduction in postoperative discomfort, but limited osteotomy and short operation time can lead to a significant decrease in postoperative swelling and pain.

**Table 3:** Results.

Case No.	1	2	3
Age (years)/sex	29/F	11/M	16/M
Angle formed by the mesiodens axis with the nasal floor	85.93°	143.5°	144.4°
Distance from the ANS (mm)	8.98	8.11	13.22
Distance to mesiodens from buccal aspect (mm)	4.71	3.70	3.84
Distance to mesiodens from palatal aspect (mm)	6.43	5.47	6.82
Relation with nasal cavity	Covered with bone	Submucosally	Covered with bone
Position regarding nasopalatine canal	In front	In front	In front
Operation time (minutes)	30	28	31
Complications	No	No	No

## Data Availability

The data generated in this study may be requested from the corresponding author.
